# The Study on Nondestructive Detection Methods for Internal Quality of Korla Fragrant Pears Based on Near-Infrared Spectroscopy and Machine Learning

**DOI:** 10.3390/foods13213522

**Published:** 2024-11-04

**Authors:** Jikai Che, Qing Liang, Yifan Xia, Yang Liu, Hongshan Li, Ninggang Hu, Weibo Cheng, Hong Zhang, Hong Zhang, Haipeng Lan

**Affiliations:** 1 College of Mechanical and Electronic Engineering, Tarim University, Alaer 843300, China; 10757232218@stumail.taru.edu.cn (J.C.); 10757232263@stumail.taru.edu.cn (Q.L.); 1075723227@stumail.taru.edu.cn (Y.X.); 18091627518@163.com (H.L.); h3330648172@163.com (N.H.); 18707364324@139.com (W.C.); 120050025@taru.edu.cn (H.Z.); 120110045@taru.edu.cn (H.L.); 2 Modern Agricultural Engineering Key Laboratory at Universities of Education Department of Xinjiang Uygur Autonomous Region, Alaer 843300, China; 120230116@taru.edu.cn; 3 Xinjiang Production and Construction Corps Key Laboratory of Utilization and Equipment of Special Agricultural and Forestry Products in Southern Xinjiang, Alaer 843300, China; 4 College of Hydraulic and Architectural Engineering, Tarim University, Alaer 843300, China

**Keywords:** Korla fragrant pear, nondestructive testing, soluble solids content, hardness, partial least squares regression

## Abstract

Quality control and grading of Korla fragrant pears significantly impact their commercial value. Rapid and non-destructive detection of soluble solids content (SSC) and firmness is crucial to improving this. This study proposes a method combining near-infrared spectroscopy (NIRS) with machine learning for the rapid, non-destructive detection of SSC and firmness in Korla pears. By analyzing absorbance in the 900–1800 nm range, six preprocessing methods—Savitzky–Golay derivative (SGD), standard normal variate (SNV), multiplicative scatter correction (MSC), Savitzky–Golay smoothing (SGS), vector normalization (VN), and min-max normalization (MMN)—were applied to the raw spectral data. uninformative variable elimination (UVE) and successive projections algorithm (SPA) were then used to extract effective wavelengths. Partial least squares regression (PLSR) models were developed for SSC and firmness based on the extracted data. The results showed that all preprocessing and wavelength-extraction methods improved model accuracy. The optimal SSC prediction model was MSC-SPA-PLSR (R = 0.93, RMSE = 0.195), and the best hardness prediction model was MSC-UVE-PLSR (R = 0.83, RMSE = 0.249). This research aids in establishing a non-destructive testing system, offering producers a rapid and accurate quality assessment tool, and provides the food industry with better production control measures to enhance standardization and market competitiveness of Korla pears.

## 1. Introduction

The Korla fragrant pear (*Pyrus sinkiangensis Yü*), a specialty fruit from southern Xinjiang, China, is highly popular for its high sugar content, crisp texture, and rich nutritional value [1,2]. Soluble solids content (SSC) and firmness are key indicators of Korla pears’ quality, playing a crucial role in ensuring optimal ripeness, taste, and quality, and significantly influencing consumer preferences [3]. During the pear’s ripening process, its main aromatic compounds are esters (such as hexyl acetate and ethyl hexanoate), while the SSC primarily consists of fructose, glucose, and sucrose. Together, these aromatic compounds and soluble solids contribute to the pear’s unique flavor [4]. The firmness of the pear is mainly determined by the polysaccharides in the cell wall, especially pectin, which undergoes changes during ripening, leading to noticeable alterations in cell structure and intercellular spaces, thereby affecting the fruit’s firmness [5]. Currently, practitioners commonly utilize refractometers and hardness testers to measure the SSC and hardness of fragrant pear, respectively. Although these methods provide quick and accurate quality information, they are destructive, inefficient, and not suitable for online detection of the internal quality of the pear, which significantly hinders the development of the Korla fragrant pear industry [6,7]. Thus, developing a highly reliable non-destructive testing method to assess the quality indicators of fragrant pear is of great significance for achieving online quality detection and grading processing of the fruit.

Technologies such as near-infrared spectroscopy [8], hyperspectral imaging [9], electrical property detection [10], and acoustic property detection [11] had all been applied to the rapid, nondestructive testing of quality indicators in fruits and vegetables, demonstrating significant potential. Among them, hyperspectral imaging showed strong detection capabilities and could capture comprehensive spectral information. However, due to its high cost, it had been challenging to transition from laboratory environments to practical orchard industry applications [12]. Electrical property detection allowed for rapid assessment of fruit quality with relatively small data volumes, but selecting the appropriate parameters was difficult as different quality indicators corresponded to different electrical parameters [13]. Acoustic property detection quickly adapted to various mediums and was suitable for online testing, but the difficulty in making fruits produce sounds through tapping, coupled with the risk of damaging the fruit if not handled properly, posed challenges [14]. In comparison, near-infrared spectroscopy was widely used for fruit quality assessment due to its low cost, simplicity of operation, and ability to simultaneously analyze multiple indicators [15]. Emanuel [16] used NIRS to detect the SSC and hardness of Ubub fruits and demonstrated that the PLSR model is feasible for predicting quality during the ripening process of Ubub fruits. Sun [17] conducted a quantitative analysis of “Yali” pears SSC using NIRS and found that the PLSR model provided a good prediction of pears SSC. Li [18] established SSC prediction models for three different pear varieties (Cuiguan, Huanghua, and Qingxiang) using NIRS combined with PLSR and found that this technology could accurately detect the SSC of different pear varieties. Fan [19] constructed a prediction model for the SSC of apples based on NIRS and PLSR, achieving good prediction results. These studies all demonstrate that the PLSR model performs well in predicting fruit quality indicators using near-infrared spectroscopy, indicating that the PLSR model is highly feasible for predicting SSC and hardness in fragrant pears. Although NIRS has shown great potential in detecting different fruit qualities, the process of acquiring absorbance information from fruits is inevitably affected by noise, baseline drift, and sample heterogeneity, which can reduce the accuracy of the model’s predictions. Research has demonstrated that data preprocessing and effective wavelength-extraction methods can effectively eliminate these interferences. Mishra [20] employed interval partial least squares to identify important wavelengths for predicting mango hardness, significantly improving prediction performance compared to PLSR without wavelength selection. Wu [21] predicted the starch content in mung beans using NIRS combined with multiple preprocessing methods, and the results indicated a significant improvement in model prediction after preprocessing. To further improve the accuracy of NIRS detection, some researchers have combined preprocessing methods with effective wavelength-extraction techniques. Wu [22] used effective wavelength-extraction methods combined with several denoising preprocessing techniques to the NIRS analysis of green apples, and the results suggested that a PLSR model based on the combination of preprocessing and effective wavelength extraction holds great potential for assessing the internal quality of green apples. Yu [23] developed a PLSR prediction model for grain quality using NIRS by combining preprocessing methods with multi-dimensional effective wavelength-extraction techniques, and the results showed a significant improvement in model accuracy. These studies indicate that integrating preprocessing with effective wavelength extraction can effectively enhance the prediction accuracy of PLSR models. However, limited research exists on applying these combined methods to predict the SSC and hardness of Korla fragrant pears.

The objective of this study is to achieve non-destructive detection of the SSC and firmness of Korla fragrant pears using near-infrared spectroscopy. This study utilized a portable near-infrared spectrometer to measure the absorbance data of pear samples in the 900–1800 nm range. Six preprocessing methods were applied in combination with two effective wavelength-extraction techniques, UVE and SPA, to simplify and remove noise from the raw spectral data. A PLSR-based prediction model for SSC and firmness of Korla fragrant pears was established, and the optimal prediction model was selected.

## 2. Materials and Methods

### 2.1. Experimental Materials

#### 2.1.1. Sampling of Korla Fragrant Pears

The pear variety is Korla fragrant pear (*Pyrus sinkiangensis Yü*). All Korla fragrant pears used in the experiment were harvested from the same pear orchard in the 10th Company of the 10th Regiment, Alaer City, First Division of the Xinjiang Production and Construction Corps (81°4′ E, 40°6′ N), with an orchard age of 16 years and a soil type of loamy soil. The pears were picked in two batches on 1 and 8 October 2023. For each batch, pears were selected with an average single fruit weight of approximately 115 ± 10 g, oval in shape, uniform in size, smooth surface, and free from pest damage. Each batch consisted of 90 pears, totaling 180 pears. During the harvest, all pickers were required to wear gloves. After harvesting, the pears were covered with foam mesh and packed with special corrugated paper fruit packaging box to avoid damage to the fragrant pears. After the fragrant pears were returned to the laboratory, the pear near-infrared spectrum data was measured immediately, the pear hardness was measured, and finally the pear SSC was measured.

#### 2.1.2. Acquisition of Spectral Data

Wash the selected pears twice with water to eliminate dust and impurities that could influence the test results. The fragrant pears were placed in an environment without wind and light, allowing them to air dry naturally. The environmental room temperature at the test site was 18 °C, with a relative humidity of 45%. Four smooth, wrinkle-free points near the equatorial line on the surface of the fragrant pear were selected as measurement points for spectral data collection. A handheld near-infrared spectrometer (NIRmagic3500, Beijing Weichuangyingtu Technology Co., Ltd., Beijing, China) was used to detect the absorbance at these points. Each measurement point was scanned three times, and the average of the three sets of data was taken as the spectral absorbance for that point. The scanning time is 3 milliseconds with 100 scans performed. The average of the spectral data from the four points was then taken as the spectral data for each fragrant pear sample.

### 2.2. Measurement of Quality Indicators

#### 2.2.1. Firmness Measurement

Uniformly peel the skin at the marked points using a peeler. Next, employ a fruit hardness tester (Aidebao GY-4, Yueqing Aidebao Instrument Co., Ltd., Zhejiang, China) to slowly and vertically insert the probe into the designated position on the pear, ensuring that the testing location corresponds with the absorbance measurement points. Cease the insertion immediately when the flesh reaches the scale line on the probe, then promptly reset the lever. The value displayed on the hardness tester at this point represents the hardness value for that location. Following each measurement, gradually rotate the arm to its original position, press the zeroing button on the instrument, and once the digital display returns to zero, proceed with the next measurement. Record the test data, compute the average of the four measurement points, and save the result, expressed in kg/cm^2^.

#### 2.2.2. Measurement of Soluble Solids Content

When measuring SSC, first calibrate the handheld refractometer (LB32T, Suwei Electronic Technology Co., Ltd., Guangzhou, China). Following calibration, evenly cut a 1 cm^3^ piece of flesh near each of the four hardness measurement points on the sample. Press the flesh with a garlic press to extract the juice, then place a drop of the juice onto the refracting prism of the refractometer, cover it with the lid, and visually read the value at the blue-white boundary line through the eyepiece. Average the data from the four points to obtain the SSC value for the sample. After each measurement, rinse the inside of the refractor prism and lid with deionized water, then wipe with a dry, clean paper towel to prevent the next measurement from being affected.

### 2.3. Spectral Data Preprocessing Methods

In this study, the near-infrared spectra of Korla fragrant pear samples were preprocessed and analyzed used six methods: Savitzky–Golay derivative (SGD), Standard Normal Variate (SNV), Multiplicative Scatter Correction (MSC), Savitzky–Golay smoothing (SGS), Vector Normalization (VN), and Min-Max Normalization (MMN). These methods were employed to reduce or eliminate noise, baseline drift, and irrelevant signal-to-noise ratio information in the sample data, thereby enhancing the accuracy of the model.

### 2.4. Extraction of Effective Wavelengths

Near-infrared spectral data typically contained a large number of wavelength variables, many of which may not provide useful information for fruit quality detection. Additionally, factors such as environmental conditions, temperature, and instrumentation can introduce complex information and noise into the spectral data. Directly using the raw spectra for model building can lead to extended modeling times, as well as reduced stability and accuracy of the predictive model. Consequently, during the initial stages of the experiment, the raw spectral data were simplified, and representative effective wavelengths were extracted to reduce model bias, thereby improving the accuracy and stability of the model predictions. In this study, SPA and UVE were employed to extract effective wavelengths from the near-infrared spectra after applying six preprocessing methods.

#### 2.4.1. Uninformative Variables Elimination

UVE (Uninformative Variable Elimination) is a feature selection algorithm used to remove variables in multivariate data that are irrelevant or noisy for model predictions [23]. Its cored principle involves multiple sampling and analysis to assess the stability and contribution of each variable, discarding unimportant ones while retaining key variables to enhance model performance. UVE is often combined with PLSR and is particularly effective in spectral analysis. This algorithm simplified the model structure, reduced computational complexity, mitigated multicollinearity, and improved the robustness and predictive accuracy of models.

#### 2.4.2. Successive Projections Algorithm

SPA (Successive Projections Algorithm) is a feature-selection algorithm designed to eliminate redundancy and multicollinearity through successive projections, selecting the most representative variables. It performed exceptionally well in spectral data processing, particularly in the analysis of high-dimensional datasets such as NIRS [24]. SPA optimized model performance and improved prediction accuracy by reducing the interdependence among variables, ensuring that the selected variables contribute maximally to the model. Additionally, it simplified the model structure and enhanced computational efficiency and robustness.

### 2.5. Modeling Method

PLSR is an advanced multivariate regression analysis method, extensively used in analyzing near-infrared spectrometer data for food, agricultural, and pharmaceutical products [24]. By integrating the independent variables of spectral data (spectral features at different wavelengths) with the dependent variables of target quality traits, PLSR thoroughly explores the intrinsic relationship between them. During this process, it systematically eliminates latent variables that fail to significantly contribute to the variance of the target variable [25]. This characteristic markedly simplifies the model’s complexity, thereby avoiding overfitting and resulting in a final model that is more streamlined, stable, and accurate in its predictions. The results are precisely aligned with real-world data, providing robust technical support for fields such as product quality control, component analysis, and rapid detection.

### 2.6. Model Evaluation Method

In this experiment, the absorbance data of fragrant pears served as the input variables for the model, while the SSC and hardness of the pears were utilized as the output variables. Data from 180 fragrant pear samples was randomly divided into a training set and a test set in a 7:3 ratio. The predictive performance of the PLSR model was assessed using the Root Mean Square Error (RMSE) and the correlation coefficient (R). Both metrics quantify the strength of the linear relationship between two variables and the precision of the predictions. Generally, a smaller RMSE value indicates a higher accuracy of the model. The correlation coefficient varies from 0 to 1, with values closer to 1 signifying a stronger correlation between the two variables and a higher model accuracy. The formulas for RMSE and the correlation coefficient are presented below:

(1) $$R=\frac{\sqrt{\sum_{i=1}^{N}{\left(P_{i}-Q_{i}\right)}^{2}}}{\sqrt{\sum_{i=1}^{N}{\left(P_{i}-\bar{Q_{i}}\right)}^{2}}}$$

(2) $$\mathrm{RMSE}=\sqrt{\sum_{i=1}^{N}\frac{{\left(P_{i}-Q_{i}\right)}^{2}}{N}}$$

In the formulas, P*_i_* and Q*_i_* represent the predicted value and the actual value for the *i*-th data point, respectively, N is the total number of samples, and $\bar{Q_{i}}$ is the mean of the actual values.

## 3. Results

### 3.1. Spectral Data Processing and Analysis of Korla Pears

**Figure 1:**
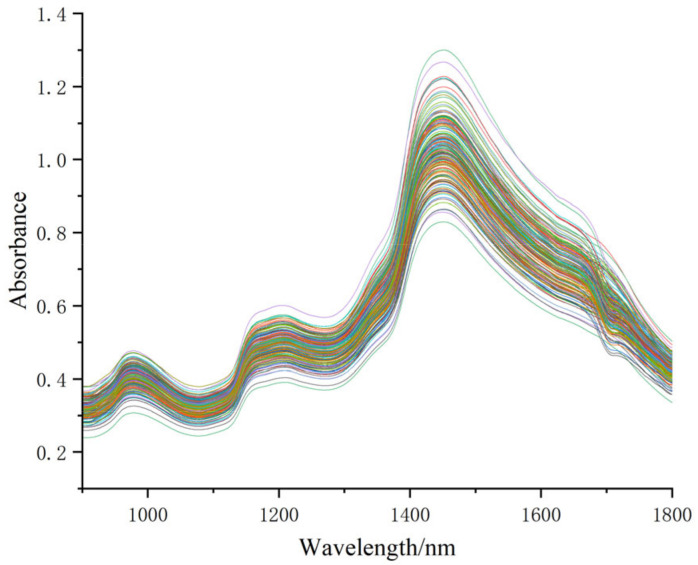
Original spectrogram of 180 fragrant pear samples.

In the process of band value variation, a wealth of information related to the quality of fragrant pears is contained. Spectral detection primarily relies on the absorption and reflection of electromagnetic waves through the overtones and combination bands of chemical bond vibrations in hydrogen-containing groups, such as C-H, N-H, and O-H. These chemical bonds are precisely the components of organic substances, and the molecular spatial arrangement and structural compactness have a significant impact on spectral changes [26]. Figure 1 shows the spectral graph of Korla fragrant pear samples within the 900–1800 nm range. Although differences exist among individual sample spectra, the overall trend remains consistent. Yu [27] used NIRS to detect the SSC of Korla fragrant pears and found absorption peaks around 950 nm, 1180 nm, and 1450 nm, with the highest peak at 1450 nm. These findings are consistent with the results of this study. The absorption peak around 1200 nm was associated with the first overtone of O-H band in water. The peak appeared at about 1450 nm and was attributed to the combination of second overtone of C-H stretching and the first overtone of O-H stretching [28]. C-H bonds are typically associated with sugars and aromatic compounds in fruits, influencing their sweetness and aroma. Fruits with high sugar content often have more C-H bonds, giving them a sweeter taste. O-H bonds are primarily found in water and alcohol compounds and are related to fruit qualities such as moisture content, acidity, and firmness. N-H and O-H bonds play a structural role in components like pectin, cellulose, and proteins within the fruit’s cell wall, where the quantity and arrangement of these bonds affect the hardness, crispness, and texture of the fruit. Therefore, near-infrared spectroscopy can effectively reflect the internal quality of fruits. Pimpen [29] used NIRS to detect the SSC of mangoes and mangosteens, finding absorption peaks similar to those of Korla fragrant pears. These absorption peaks are also related to the overtones of chemical bonds. This shows that the specific chemical composition and content of different fruits have a similar effect on spectral characteristics, making near-infrared spectroscopy suitable for quality detection across various fruits.

**Figure 2:**
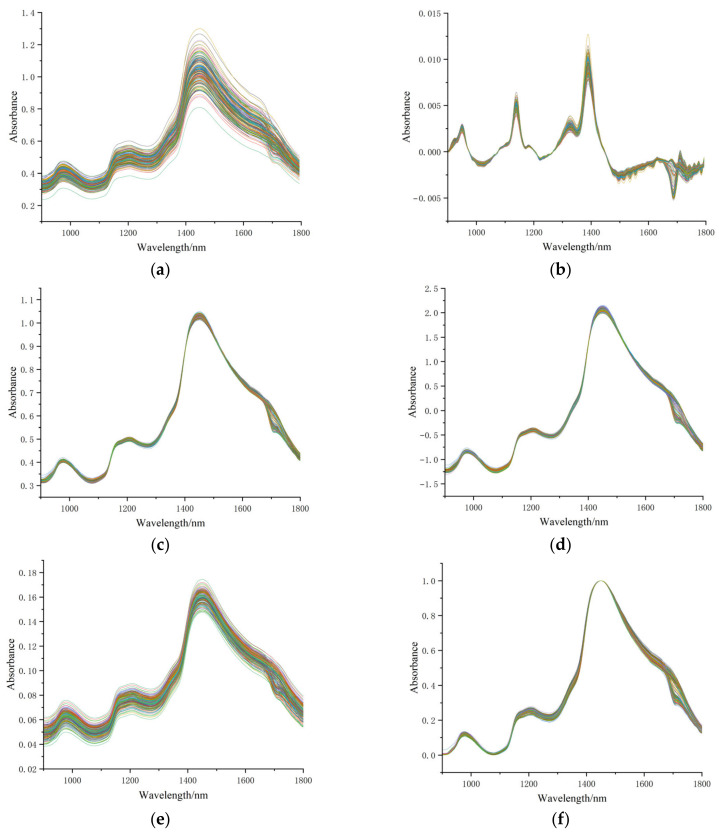
Spectral characteristic changes of 180 Korla pear samples after six preprocessing methods ((**a**): SGS, (**b**): SGD, (**c**): MSC, (**d**): SNV, (**e**): VN, (**f**): MMN).

It can be observed from Figure 1 that NIRS bands are relatively broad, with significant spectral overlap, making it difficult to analyze the corresponding bands accurately. To improve the quality of spectral data and enhance the accuracy and reliability of the analytical model, the raw spectral data were subjected to six preprocessing methods to obtain new spectra for subsequent model building, as shown in Figure 2. SGS reduces noise and maintains the peaks and troughs, making the curve smoother while preserving the original spectral characteristics and reducing random noise. SGD, as a derivative method, amplifies subtle features in the data, making the spectral peaks more distinct and enhancing the details that are difficult to observe in the raw data. MSC flattens the spectral curves, minimizing the variation in absorbance caused by scattering and improving consistency across different samples. SNV standardizes and mean-centers each data point, reducing scattering differences between samples and improving the accuracy of the model. VN enhances minor spectral features that may be obscured by larger variances, facilitating the detection of meaningful differences. MMN maps the original data through linear transformation to a new range, eliminating the impact of different feature scales, enabling more uniform analysis and modeling, and improving algorithm performance. Zhu and Bai [30,31] used preprocessing methods such as SGS, SNV, SGD, MSC, and MMN to preprocess the near-infrared spectral bands of corn seeds and wine, and the research findings were similar to those in this study. Compared to the raw spectrum, SGS reduces some of the random noise present in the original spectrum, although the difference is not significant. The application of derivatives in SGD amplifies the inflection points that represent quality-related elements of the fruit, thereby enhancing model accuracy. MSC, SNV, and VN show similar spectral changes, differing mainly in absorbance values. All three preprocessing methods effectively eliminate scattering phenomena in the spectral data, focusing the internal spectral information within samples. After MMN processing, the spectral data around the trough at 1080 nm and the peak at 1480 nm become highly concentrated. However, due to irregular changes in the maximum and minimum spectral values, spectral data dispersion occurs at 1750 nm.

### 3.2. Prediction of Pear Quality Using Full-Spectrum Data

#### 3.2.1. SSC Prediction After Full-Spectrum Preprocessing

PLSR prediction models were developed for the absorbance data after six types of preprocessing, as well as for the raw absorbance data, with the R and RMSE of the models subsequently compared. The prediction results are presented in Table 1. The PLSR prediction model based on the raw spectra recorded an R of 0.74 and an RMSE of 0.398. Compared to the unprocessed model, all six preprocessing methods enhanced prediction accuracy. Among these, the PLSR model utilizing MMN-processed spectra achieved the highest R = 0.78 and the smallest RMSE = 0.337, yielding the best prediction performance.

#### 3.2.2. Firmness Prediction After Full-Spectrum Preprocessing

PLSR prediction models were developed for the absorbance data after six types of preprocessing, as well as for the raw absorbance data, with subsequent comparisons of the R and RMSE. The prediction results are presented in Table 2. The prediction model based on the raw spectra recorded an R of 0.56 and an RMSE of 0.344. Compared to the unprocessed model, all six preprocessing methods enhanced prediction accuracy. Among these, the PLSR model employing spectra processed with the SG convolution derivative method achieved the highest R = 0.67 and the lowest RMSE = 0.28, yielding the best prediction performance.

As shown in Table 1 and Table 2, various preprocessing methods have improved the accuracy of the PLSR prediction model based on the raw spectra, further enhancing the PLSR model’s ability to predict fruit quality. However, although the addition of preprocessing methods yields better numerical performance than without preprocessing, the prediction results still fall short of the expected targets, indicating the need for further data optimization.

### 3.3. Quality Prediction of Fragrant Pears Based on Effective Wavelength Extraction

To further enhance the accuracy of the PLSR model, this study incorporated two effective wavelength-extraction methods (UVE and SPA) based on several preprocessing techniques. The combination of preprocessing and effective wavelength extraction helps eliminate redundant and outlier spectral bands, simplifying the model’s complexity to improve processing efficiency and prediction accuracy. UVE improves prediction performance and model robustness by analyzing the contribution of spectral variables and removing variables that are insignificant or contain excessive noise. Meanwhile, SPA effectively eliminates redundant information and collinearity issues, reducing irrelevant or duplicate spectral variables in the model, simplifying its structure, and lowering the risk of overfitting, thereby enhancing prediction accuracy.

#### 3.3.1. SSC Prediction Using Extracted Effective Wavelengths

As presented in Table 3, the analysis of PLSR prediction models for SSC in Korla fragrant pears, developed by integrating various preprocessing methods with effective wavelength-extraction techniques, demonstrates that this combination enhances model accuracy. Without preprocessing, the PLSR model employing SPA for wavelength extraction recorded an R value of 0.86 and an RMSE of 0.267, whereas the model utilizing UVE for wavelength extraction registered an R value of 0.83 and an RMSE of 0.323. The PLSR models that combined preprocessing with effective wavelength-extraction methods outperformed those employing either a single preprocessing or a single wavelength-extraction method. When SPA served as the wavelength-extraction method, all models post-preprocessing exhibited R values exceeding 0.9 and RMSE values below 0.227. The PLSR model with MSC preprocessing attained the highest R value of 0.93 and the lowest RMSE value of 0.195. When UVE functioned as the wavelength-extraction method, all models post-preprocessing recorded R values above 0.86 and RMSE values below 0.269. The PLSR model incorporating MMN preprocessing achieved the highest R value of 0.91 and a relatively low RMSE value of 0.212.

#### 3.3.2. Firmness Prediction Using Extracted Effective Wavelengths

As presented in Table 4, an analysis of PLSR hardness prediction models for Korla fragrant pears, developed by integrating various preprocessing methods with effective wavelength-extraction techniques, demonstrates that diverse combinations of these methods enhance the accuracy of PLSR models. Without preprocessing, the PLSR model employing SPA for wavelength extraction recorded an R value of 0.71 and an RMSE of 0.274, whereas the model utilizing UVE registered an R value of 0.78 and an RMSE of 0.287. When comparing PLSR models that combine preprocessing with effective wavelength extraction against those using a single method, the combined approach significantly improves prediction accuracy. Specifically, with SPA as the wavelength-extraction method, all models, except for the one employing SNV preprocessing, exhibited R values above 0.72 and RMSE values below 0.251. The PLSR model utilizing SG convolution derivative preprocessing achieved the highest R value of 0.78 and a relatively low RMSE of 0.244. Similarly, when UVE functioned as the wavelength-extraction method, all models recorded R values above 0.78. Except for the models employing SGS and VN preprocessing, RMSE values were all below 0.277. The PLSR model incorporating MSC preprocessing attained the highest R value of 0.83 and an RMSE of 0.247, demonstrating superior prediction performance.

The comparison of model results indicates that in the prediction models for SSC in Korla fragrant pears, the MSC-SPA-PLSR model achieved the best prediction performance, with the highest R value and the lowest RMSE (R = 0.93, RMSE = 0.195). For the prediction models of hardness in Korla fragrant pears, the MSC-UVE-PLSR model provided the best prediction performance, with the highest R value and a relatively low RMSE (R = 0.83, RMSE = 0.247).

## 4. Discussion

In this study, NIRS was employed to measure the absorbance of Korla fragrant pears. Six preprocessing and two effective wavelength-extraction methods were integrated to develop PLSR-based prediction models for the SSC and hardness of the pears. The optimal predictive model for SSC was MSC-SPA-PLSR, achieving an R value of 0.93 and an RMSE of 0.195. The superior model for predicting hardness was MSC-UVE-PLSR, achieving an R value of 0.83 and an RMSE of 0.247.

Other researchers have also used non-destructive testing techniques combined with machine learning to predict pear quality. Yu [32] utilized hyperspectral technology combined with the SAE-FNN model to detect the SSC of Korla fragrant pears and developed a prediction model (R = 0.93, RMSE = 1.81). Fang [33] used dielectric spectroscopy combined with the ELM model to predict the SSC and firmness of Korla fragrant pears. The optimal prediction accuracy for SSC was (R = 0.876, RMSE = 0.330), and the optimal prediction accuracy for firmness was (R = 0.56, RMSE = 0.389). In this study, near infrared spectroscopy combined with the PLSR model was used to predict SSC and hardness of Korla pears. The optimal prediction accuracy of SSC and hardness was (R = 0.93, RMSE = 0.195), and the optimal prediction accuracy of hardness was (R = 0.83, RMSE = 0.247). Compared with other techniques, this study obtained better prediction results. The higher accuracy of near-infrared spectroscopy (NIRS) is primarily due to its ability to directly detect characteristic absorption peaks closely related to the properties of organic molecules. These absorption peaks correspond to the vibrations and overtones of bonds such as C-H, O-H, and N-H, which are widely present in the main components of fruits, such as sugars, acids, and water. By identifying these characteristic absorption peaks, NIRS can capture specific information about the internal composition of the fruit, such as variations in sugar concentration, water content, and fiber structure, indirectly indicating the SSC and firmness of the fruit. This information is crucial to assessing fruit quality. Liu [34] employed NIRS to establish a least squares support vector machine (LSSVM) model for predicting the SSC and firmness of fragrant pears, achieving SSC predictions of (R = 0.88, RMSE = 1.738) and firmness predictions of (R = 0.826, RMSE = 2.199). The PLSR model used in this study outperformed the LSSVM model, indicating that PLSR exhibits better adaptability for predicting SSC and firmness of Korla fragrant pears using NIRS. This may be because the computational complexity of LSSVM increases with higher data dimensions, which can reduce efficiency and accuracy when handling large data sets. In contrast, PLSR performs well when the number of variables far exceeds the sample size and maintains robust performance even in the presence of outliers. Zhang [35] used the SPA and UVE algorithms to extract effective wavelengths from hyperspectral data of Dangshan pears and established a PLSR-based sugar content prediction model. The model’s prediction accuracy was (R = 0.86, RMSE = 0.384) with SPA, and (R = 0.87, RMSE = 0.399) with UVE. Liao [36] and colleagues applied SGS, MSC, and Gaussian filtering (GFS) methods to preprocess NIRS data of Gong pears and built a PLSR model for sugar content prediction, achieving optimal model accuracy of (R = 0.9, RMSE = 0.371). These findings are consistent with the results of this study, confirming that combining preprocessing and effective wavelength extraction improves model accuracy compared to using a single preprocessing or extraction method. In this study, the SSC prediction accuracy was higher than the firmness prediction accuracy, which aligns with the findings of Wang [37] and Lu [38]. This may be because sugar content measurements primarily rely on the absorption or refraction characteristics of sugar molecules inside the fruit, which are relatively stable and easy to measure, yielding higher accuracy. In contrast, firmness measurement involves the mechanical properties of the fruit, which are influenced by various complex factors such as cell structure, moisture content, and ripeness, resulting in greater measurement deviations and affecting model accuracy.

Additionally, the study will explore the applicability of NIRS in non-destructive fruit-quality testing, aiming to develop relevant software and instruments to meet the high-precision, high-efficiency, non-destructive testing needs for characteristic fruits in Xinjiang, China, thereby establishing a more comprehensive fruit-quality detection system.

## 5. Conclusions

This study measured the absorbance of Korla fragrant pears in the 900–1800 nm range using a near-infrared spectrometer. Combining multiple preprocessing methods and effective wavelength-extraction techniques, a PLSR-based prediction model for the soluble solids content (SSC) and firmness of Korla fragrant pears was developed to enable non-destructive quality detection. The analysis of spectral variations reveals that the absorbance data gradually increases within the 900–1480 nm range, while it gradually decreases in the 1480–1800 nm range. Distinct peaks are observed at 980 nm, 1200 nm, and 1480 nm, with the highest peak appearing at 1480 nm. Compared to raw spectral data, the PLSR prediction models constructed with six preprocessing methods (SGS, SG, MSC, SNV, VN, MMN) and two effective wavelength-extraction methods (SPA and UVE) demonstrated enhanced accuracy. The optimal SSC prediction model was MSC-SPA-PLSR, achieving an R value of 0.93 and an RMSE of 0.195, while the best hardness prediction model was MSC-UVE-PLSR, with an R value of 0.83 and an RMSE of 0.247. In the next phase of the research, the data processing methods will be optimized, and different algorithms will be used to enhance the accuracy of the prediction models. This will enable the detection of quality in various fruits and can also be extended to quality control of other agricultural products, thereby improving the efficiency and quality of the entire agricultural production and distribution system, resulting in significant commercial benefits.

## Figures and Tables

**Table 1:** Prediction results of SSC of fragrant pears after pretreatment.

Pretreatment Method	Training Set		Test Set	
	R	RMSE	R	RMSE
No preprocessing	0.84	0.314	0.74	0.398
SGS	0.89	0.272	0.76	0.366
SGD	0.89	0.273	0.76	0.346
VN	0.86	0.303	0.75	0.385
MMN	0.88	0.295	0.78	0.337
SNV	0.84	0.325	0.74	0.388
MSC	0.86	0.305	0.74	0.320

**Table 2:** Prediction results of hardness of fragrant pears after pretreatment.

Pretreatment Method	Training Set		Test Set	
	R	RMSE	R	RMSE
No preprocessing	0.77	0.295	0.56	0.344
SGS	0.78	0.274	0.57	0.302
SGD	0.82	0.267	0.67	0.28
VN	0.72	0.307	0.58	0.317
MMN	0.77	0.311	0.58	0.318
SNV	0.78	0.300	0.67	0.307
MSC	0.79	0.287	0.59	0.296

**Table 3:** Prediction results of the SSC of fragrant pears after effective wavelength extraction.

Pretreatment Method	Effective Wavelength-Extraction Method	Training Set		Test Set	
		R	RMSE	R	RMSE
No preprocessing	SPA	0.89	0.245	0.86	0.267
	UVE	0.84	0.314	0.83	0.323
SGS	SPA	0.95	0.201	0.93	0.220
	UVE	0.95	0.195	0.89	0.211
SGD	SPA	0.95	0.194	0.91	0.209
	UVE	0.95	0.217	0.91	0.219
VN	SPA	0.95	0.191	0.91	0.227
	UVE	0.91	0.254	0.88	0.269
MMN	SPA	0.95	0.190	0.90	0.207
	UVE	0.93	0.209	0.91	0.212
SNV	SPA	0.95	0.208	0.91	0.212
	UVE	0.92	0.229	0.88	0.241
MSC	SPA	0.96	0.194	0.93	0.195
	UVE	0.91	0.249	0.86	0.255

**Table 4:** Prediction results of the hardness of fragrant pears after effective wavelength extraction.

Pretreatment Method	Effective Wavelength-Extraction Method	Training Set		Test Set	
		R	RMSE	R	RMSE
No preprocessing	SPA	0.84	0.231	0.71	0.267
	UVE	0.84	0.282	0.78	0.287
SGS	SPA	0.85	0.221	0.72	0.241
	UVE	0.83	0.310	0.78	0.325
SGD	SPA	0.89	0.227	0.78	0.244
	UVE	0.85	0.249	0.8	0.277
VN	SPA	0.87	0.211	0.75	0.220
	UVE	0.84	0.302	0.8	0.31
MMN	SPA	0.88	0.231	0.72	0.251
	UVE	0.83	0.280	0.79	0.244
SNV	SPA	0.83	0.291	0.71	0.305
	UVE	0.87	0.237	0.78	0.240
MSC	SPA	0.88	0.226	0.72	0.237
	UVE	0.87	0.245	0.83	0.247

## Data Availability

The original contributions presented in the study are included in the article, further inquiries can be directed to the corresponding author.
